# Spatiotemporal Evolution and Emergence of Frontier Malaria Mosaics in the Brazilian Amazon

**DOI:** 10.21203/rs.3.rs-9919747/v1

**Published:** 2026-06-09

**Authors:** Alisson Barbieri, Reinaldo Santos, Getúlio Domingues, Mark Janko, Richard Moreira, William Pan

**Affiliations:** Universidade Federal de Minas Gerais; Universidade Federal de Minas Gerais; Universidade Federal de Minas Gerais; University of Buffalo; Universidade Federal de Minas Gerais; Duke University

**Keywords:** Frontier malaria mosaics, Brazilian Amazon, population mobility, garimpo, indigenous

## Abstract

Malaria is a major global health problem, including the Brazilian Amazon region. The theory of frontier malaria—linking agricultural expansion, migration, and road networks to altered land cover and disease transmission—is increasingly inadequate for contemporary Amazonian frontiers. Instead, we propose the concept of frontier malaria mosaics, reflecting diverse occupational profiles, mobility patterns, rural-urban connectivity, and land uses. As malaria declines in the Amazon, transmission becomes highly focal, clustering in localized areas with distinct characteristics. Using locality-level data from Brazil’s Malaria Surveillance System and a novel method to identify nearly all case locations, we analyze spatiotemporal evolution via parasite species, infection origin, land use, settlement patterns, and sociodemographics. Annualized indicators enable cross-period comparisons. Through cluster regionalization, we reveal distinct low- and high-transmission mosaics, each shaped by successive government policies. This approach overcomes key data limitations and demonstrates how local spatial configurations scale into regional patterns over time.

## INTRODUCTION

Locally transmitted malaria in Brazil is almost entirely confined to the Amazon, accounting for 99–100% of cases [1]. Despite government efforts to eliminate the disease [2] and successful initiatives in neighboring Amazonian countries [3, 4], control and elimination remain challenging and involve, among other factors, improving active surveillance, timely diagnosis and treatment especially in remote and international border areas [4–6].

In Amazonian literature, frontier describes a sparsely populated region with nascent economic potential, attracting external settlement linked to diverse land uses, including agriculture, ranching, logging, mining (both artisanal and industrial), urban infrastructure and large-scale projects like roads and dams. Frontiers often evolve in cycles or phases driven by demographic, environmental, and political shifts [7]. They may be abandoned after a short-term extractive boom [8] or see their economic activities contract. [9]. Cycles begin with initial occupation, marked by extensive deforestation. It expands through new land incorporation and rising population density, often from in-migration, culminating in a consolidated post-frontier stage integrated into broader markets [7, 10–16].

Frontier expansion [17, 18] has been associated with weakened environmental legislation and deforestation [1, 19], poverty and inequality [20], violence [21, 22], and high malaria incidence [17, 18]. Regarding this last, the *theory of frontier malaria* emerged as an expression of socio-epidemiological consequences of frontier expansion, with the interplay of agriculture-based human settlement, road opening, environmental change associated with deforestation and changing vector’s habitat is associated with high malaria incidence [5, 17, 18, 23–29]. Historically, it refers to the expansion of agricultural frontier, mainly in the 1970–1980s, with large population inflows from of Brazi’s Southern and Northeastern regions to the Amazon, facilitated by road expansion and official and private settlement projects [26, 30, 31]. As for the concept of frontier, frontier malaria presents a temporal pattern summarized in three phases. First, the epidemic phase, marked by intense outbreaks observed for about 3 years after initial settlement. Outbreaks result from a combination of factors that include deforestation, which creates or expands breeding habitats of the main local malaria vector, *Anopheles darlingi* [37], close to colonists’ ramshackle houses in the fringes of the rainforest [38–40], lack of acquired immunity among most of the settlers, precarious habitat conditions that offered no protection against mosquitoes, and lack of adequate knowledge of the disease, among others. Second, the transition phase, characterized by gradual declines in malaria transmission observed over the following years, as the farming settlement becomes consolidated, with less environmental changes, better structured communities, improved housing, and better access to healthcare [35, 41, 42]. Finally, in the endemic phase, malaria transmission reaches lower and stable levels. In this setting, P. falciparum typically predominates in newly opened settlements in areas of rapid ecological change (including in vectors’ habitat) due to human population migration, being progressively replaced by P. vivax in later stages [26, 32].

However, prior conceptualizations of frontier malaria focused on only certain localities or regions, and mostly on associations between malaria, migration and the expansion of roads and agriculture. The theory of frontier malaria assumes a closed system independent from exogenous policies, describing an autonomous process of transmission phases. This assumption is flawed, as real-world systems are not closed, rendering the theory of limited explanatory relevance—particularly for malaria control. In practice, government policies shape livelihood decisions (e.g., land use, mobility, rural-urban connectivity) and directly address malaria’s underlying and proximate causes (e.g., through epidemiological surveillance and interventions). Policies across all sectors, not just health, are key drivers of transmission dynamics. The theory remains disconnected from these levers, encouraging an overemphasis on links between deforestation, colonization, mining, and migration while neglecting direct analysis of how government policies shape proximate drivers of malaria transmission.

In order to overcome these limitations, recent studies on frontier malaria have accounted for diverse forms of human mobility and circulation (besides migration) and rural-urban connectivity [19, 29, 33–35], livelihoods diversification associated with specific occupational profiles [27] and sociodemographic characteristics [9, 35], higher complexity and juxtaposition of land use strategies, including roads, dams, mining and agricultural settlements and their context-specific patterns of environmental change and deforestation [25, 28, 29, 34]. Policy shifts affecting environmental protection and health and thus malaria transmission have also increasingly been discussed [1, 19, 36]. Malaria reduction prior to 2016 in Brazil is associated with the enforcement of malaria control programs such as the National Malaria Prevention and Control Program (PNCM) created in 2003 and the program for Elimination of P. falciparum in 2015 [2, 37]. On the other hand, recent increases in indigenous and mining areas [19] and higher P. falciparum incidence linked to cases imported from neighboring countries like Venezuela [38], Peru and Colombia [39] has followed relaxed enforcement of environmental legislation, particularly in indigenous and protected areas..

Another limitation in the theory of frontier malaria refers to the spatial scale. While previous studies often studied frontier malaria at a municipal level in Brazil [27, 40], frontier malaria dynamics operate at a local scale, influenced by contextual characteristics [26, 35]. This is evident in interconnected areas by mobility like mining sites (garimpos), indigenous territories, and rural-urban zones [19, 34, 38]. In consolidated frontiers, greater connectivity with urban economies drives resource-intensive land use, such as mining expansion within the Yanomami Indigenous Territory [19]. Frontier malaria also exhibits distinct population selectivity, disproportionately affecting specific occupational groups (e.g., garimpo workers, known as garimpeiros), demographics (e.g., young males), ethnicities (e.g., indigenous populations interacting with outsiders) [19], and colonist settlements with poor housing and limited healthcare access [19, 26, 41–43]. For instance, indigenous groups experience more severe malaria outcomes compared to other race/ethnic groups, including higher parasitemia and a greater burden of P. falciparum in children [44].

To understand contemporary Amazonian malaria dynamics, we must look beyond the traditional theory of frontier malaria and its focus on agricultural expansion. We introduce the theory of a frontier malaria mosaic, which expands this framework to incorporate the complex interplay of human mobility, rural-urban connectivity, diverse livelihoods, and evolving political and socioeconomic contexts. Rather than a single, uniform Amazon frontier, multiple distinct frontier mosaics may emerge over time with distinguishing characteristics and in specific Amazonian settings and populations. As malaria cases in the Amazon have diminished but becoming increasingly focal over time and often clustered in specific, localized land-use areas, empirical evidences on the mechanisms behind the emergence and evolution of mosaics representing transmission dynamics must be obtained mainly from finer-scale levels.

We propose the theory of frontier malaria mosaics to evaluate key characteristics of malaria trends over the past 17 years (2007–2023) in the Brazilian Amazon. This is a novel contribution for three reasons. First, we adopt a bottom-up approach, using locality-level data from the Brazilian Malaria Surveillance System (SIVEP-Malaria) to identify local spatial configurations of frontier malaria, including interactions between localities of residence and likely malaria infection and with distinct land uses, and trace how they scale up into regional patterns over time. To the extent of our knowledge, and except for a regional study focusing on the Northern Brazilian Amazon [19], no other studied undertook an analysis focusing on malaria dynamics at the locality level, particularly covering all the Amazon. Second, we developed a novel method to spatially identify nearly all malaria case localities, overcoming a key limitation of the SIVEP-Malaria data. Third, we analyze the spatiotemporal evolution of frontier malaria through variables including parasite species, infection origin (autochthonous or imported), land use, settlement patterns, and the sociodemographic profiles of infected individuals.

We analyze how policies associated with successive federal government terms help explaining the consolidation of heterogeneous malaria frontiers into distinct mosaics. We focus on federal government policies for two reasons. First, 48.5% of the Amazon is federally protected, including all Indigenous lands and most conservation units [45]. Evidence discussed earlier in this paper indicates that malaria transmission across federal government periods has been shaped by varying enforcement of environmental laws that govern intrusion and occupation of protected areas. Second, the federal government is the primary agent responsible for malaria control and surveillance programs, including financing, health facilities, and treatment [2].

## RESULTS

### Descriptive analysis.

**Figure 1:**
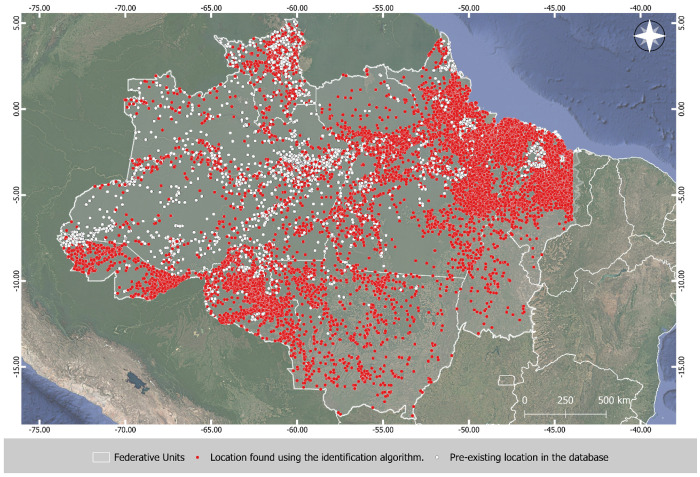
Spatial distribution of locations found using the identification algorithm

Table 1 and Fig. 1 demonstrate a substantial improvement in the spatial identification of localities in the SIVEP-Malaria database. Our method increased spatial coverage from 7.1% to 98.4% of all localities, accounting for 99.4% of the 3,601,061 malaria cases from 2007–2023. Coverage increased significantly across all land-use types, most notably for *garimpos* (91.4% of localities and 30.8% of cases in 2007–2023), rural areas (93.6% of localities, 38.4% of cases), and urban areas (80.3% of localities, 19.0% of cases).

**Figure 2:**
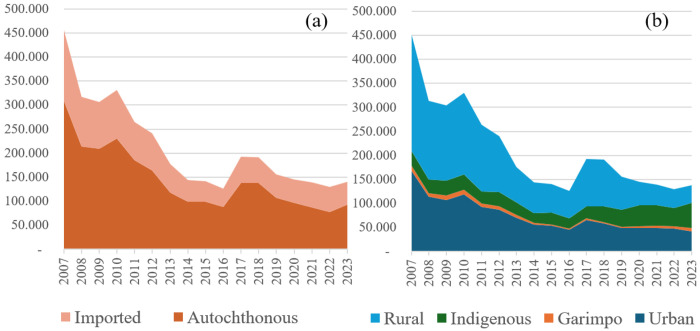
Evolution of malaria cases, considering a) imported* and autochthonous cases, b) types of localities of residence, Brazilian Amazon, 2007-2023 Source: Elaborated from SIVEP-Malaria dataset * Individuals living in a locality in Brazil who had likely infection in a different locality, in Brazil or abroad.

Figure 2 shows the temporal dynamics of malaria in the Brazilian Amazon. While the relative proportion of imported cases grew over time, autochthonous cases remained consistently higher in absolute numbers from 2007–2023 (Fig. 2a). After a massive decline between 2007–2016 mainly in rural and urban malaria, in a great extent due to the enforcement of public malaria control programs [2, 37], a surge from 2016–2019 was primarily driven by rural residents, increasing autochthonous transmission. The distribution of cases by land use also shifted (Fig. 2b). Initially dominant, rural malaria later decreased, with urban and indigenous cases reaching similar levels. The consistently low proportion of *garimpo* cases is likely due to miners residing in urban areas—explaining the high imported cases in Table 1—and potential underreporting linked to illegal activities.

### Space-time density of malaria cases and Net Malaria Mobility (NMM).

The evolution of malaria across land uses (Fig. 2) suggests that contextual factors, including governmental shifts, may be a likely influence. This aligns with the concept of the Amazon harboring multiple, heterogeneous malaria frontiers rather than a single, uniform one, as described in prior frontier malaria research in the Amazon.

**Figure 3:**
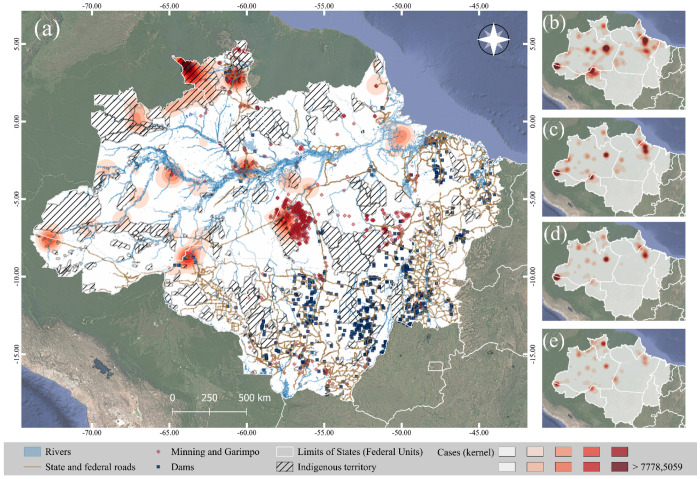
Density of malaria cases in the Brazilian Legal Amazon by Federal Government Periods– 2007 to 2023* * Panels: (a) President Lula 3^rd^ term (Lula III), 2023; (b) President Lula 2^nd^ term (Lula II), 2007-2010; (c) President Dilma 1^st^ term (Dilma I), 2011-2014; (d) President Dilma 2^nd^ term, impeached, and President Term (2015-2018); (e) President Bolsonaro, 2019-2022. Source: Elaborated from SIVEP-Malaria (2023) and IBGE (2023) datasets

**Figure 4:**
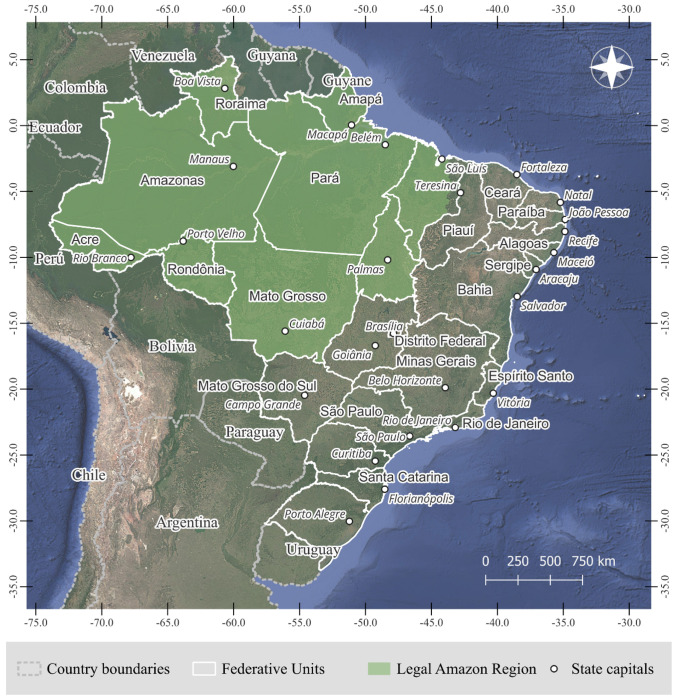
Brazil: Limits of the legal Amazon, Federation Units and state capitals

Figure 3 illustrates the spatiotemporal evolution of malaria from 2007–2023, detailing the periods associated with the second term of President Lula, 2007–2010 (Fig. 3b), first term of President Dilma, 2011–2014 (Fig. 3c), second term of President Dilma and, after her impeachment, President Temer, 2015–2018 (Fig. 3d), President Bolsonaro, 2019–2022 (Fig. 3e) and the third term of President Lula, 2023 (Fig. 3a). Results show a stable distribution of cases, with higher densities in areas of significant anthropogenic activity—such as infrastructure projects, mining, and *garimpos* — and their connected urban centers, including states and capitals [46] (Fig. 4). It is noteworthy that the intensity of hotspots has decreased, validating the reduction in the number of cases throughout the Brazilian Amazon (Fig. 2). However, other significant hotspots emerge over time, such as in the northernmost part of the Amazon (State of Roraima) between 2019 and 2022 and that includes the Yanomami Indigenous Territory.

**Figure 5:**
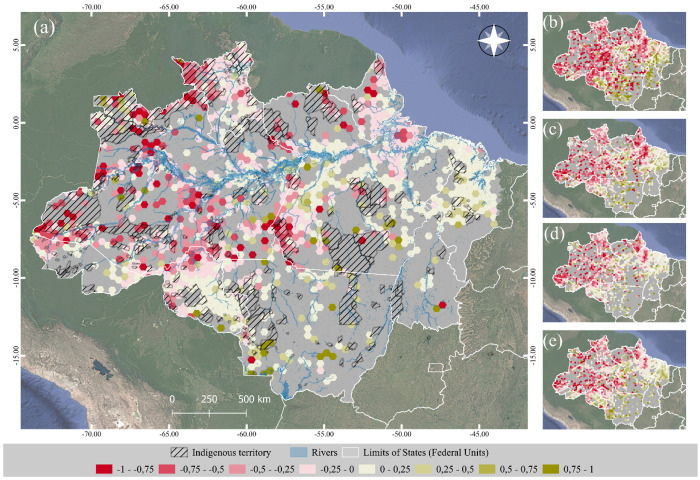
Identification of areas with predominance of autochthonous cases (red) and imported cases (yellow) in the Legal Amazon - Net Malaria Mobility Rate (NMR), 2007 to 2023* * Panels: (a) President Lula 3^rd^ term (Lula III), 2023; (b) President Lula 2^nd^ term (Lula II), 2007-2010; (c) President Dilma 1^st^ term (Dilma I), 2011-2014; (d) President Dilma 2^nd^ term, impeached, and President Term (2015-2018); (e) President Bolsonaro, 2019-2022. Source: Elaborated from SIVEP-Malaria (2023) and IBGE (2023) datasets

Figure 5 maps the of Net Malaria Mobility (NMM) to differentiate between autochthonous (red) and imported (yellow) cases over time. Unlike the stable overall distribution of malaria cases, the NMM reveals significant epidemiological shifts. While some regions, such as central and eastern Amazonia, experienced an increase in imported cases, historically autochthonous areas also showed growing importation over time.

Notably, in the Yanomami Indigenous Territory (YIT) in Roraima state (Fig. 4), mining activity intensifying since 2019 has exacerbated local transmission (indicated by high negative NMM, reflecting autochthonous cases) while simultaneously driving a rise in imported cases (high positive NMM) in nearby urban areas—particularly in Boa Vista, the state capital. This emergent pattern is likely attributable to mobility networks connecting urban centers and mining areas (*garimpos*) [19]. A similar dynamic appears in the northeast (Marajó Island in Pará state, Fig. 4) and in the south/southwest regions (especially along the borders of Acre and Rondônia with Amazonas state, Fig. 4), where clusters of high positive and high negative NMM have been in close proximity since 2011. In Rondônia specifically, the co-occurrence of these clusters has been linked to population movement between residential areas and the Jirau hydropower dam reservoir, as well as between urban and rural zones [47]. These cases—where malaria dynamics appear closely tied to human mobility—highlight persistent challenges for local malaria control. In contrast, over time, other clusters in the south/southwest and northeast show a growing predominance of imported malaria (positive NMM).

### Spatiotemporal Heterogeneity of the Frontier Malaria.

**Figure 6:**
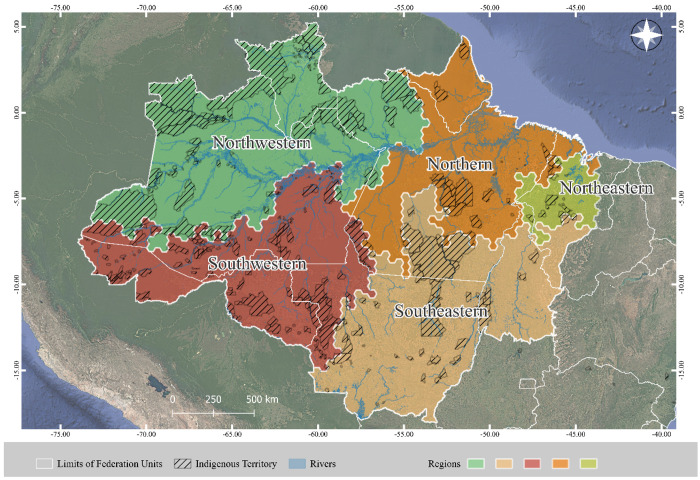
Frontier malaria mosaics in the Brazilian Amazon – 2007 to 2023 Source: Elaborated from SIVEP-Malaria (2023) dataset

Based on the previous discussion and in order to map the heterogeneous and temporally dynamic malaria frontier, we applied the SKATER algorithm. This method grouped grid cells into mosaics based on their similarity across multiple standardized variables representing key socioenvironmental aspects defining the frontier malaria as discussed in the background section. These variables, measured by Euclidean distance (see the supplementary material, Table S1), are: i) type of locality of infection (land uses representing the juxtaposition between human settlements, economic activities and specific ways of mobilizing natural resources), ii) the percentage of autochthonous cases (role of human mobility or immobility in the diffusion of malaria across space), and iii) proportion of infection by *P. falciparum* (parasitology as a marker of rapid ecological change and deforestation, with change in vectors’ habitat, due to human mobility in initial phases of frontier setttlement). These variables are available in the SIVEP-Malaria database and serve as spatial characteristics of the disease’s behavior. Other variables either lack a high degree of completeness across all years (e.g., race/color) or are more closely associated with the stage of demographic transition than with strictly epidemiological characteristics (e.g., age); therefore, they were not included in the regionalization model. It resulted in five mosaics: Northwestern (NWFM), Southeastern (SEFM), Southwestern (SWFM), Northern (NOFM), and Northeastern (NEFM) (Fig. 6).

Results show that NWFM and SWFM have the highest malaria infection in indigenous localities, with SWFM sharing with NOFM the highest rural and autochthonous malaria. NWFM follows as the third mosaic of highest rural and autochthonous malaria, distinguishing from SWFM and NOFM for a higher balance between indigenous and rural malaria. NEFM and SEFM distinguish by a more balanced distribution of malaria cases between urban, *garimpo* and rural localities, with SEFM having the lowest autochthonous malaria between all mosaics and NEFM the highest participation of localities with incidence of *P. falciparum*.

**Figure 7:**
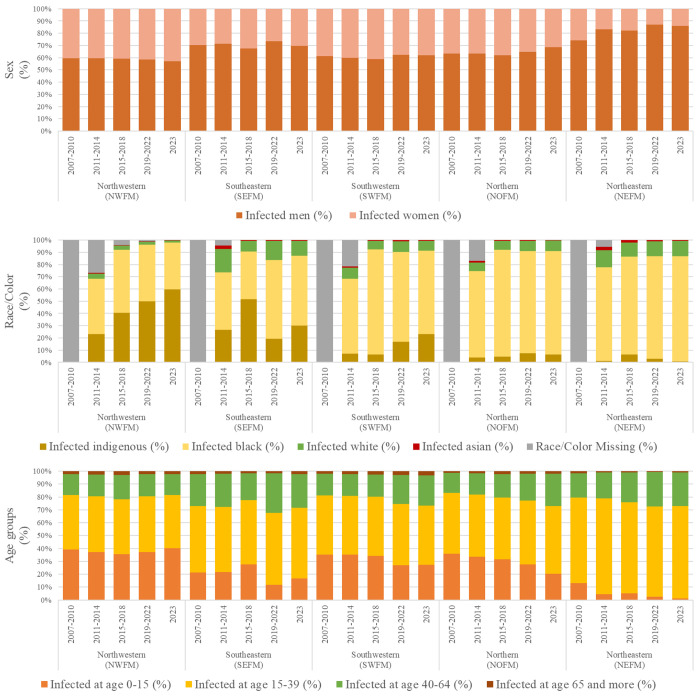
Malaria cases and Demographic characteristics* of frontier malaria mosaics by governmental periods** *Race “black” includes mixed black and white. Data differs statistically by region, period, and dimension analyzed. The tests are available in Table S4. ** Panels: (a) President Lula 3^rd^ term (Lula III), 2023; (b) President Lula 2^nd^ term (Lula II), 2007-2010; (c) President Dilma 1^st^ term (Dilma I), 2011-2014; (d) President Dilma 2^nd^ term, impeached, and President Term (2015-2018); (e) President Bolsonaro, 2019-2022. Source: Elaborated from SIVEP-Malaria (2023) dataset

**Figure 8:**
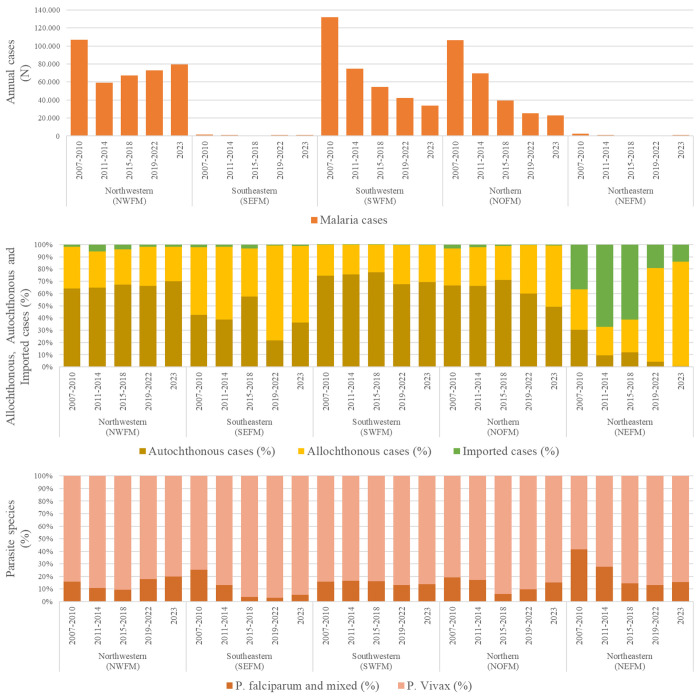
Source: Elaborated from SIVEP-Malaria (2023) dataset Epidemiological characteristics* of frontier malaria mosaics by governmental periods** *“Allochthonous” refers to all malaria cases in a locality/community of infection different from residence, excluding imported (other country). Data differs statistically by region, period, and dimension analyzed. The tests are available in Table S4. ** Panels: (a) President Lula 3rd term (Lula III), 2023; (b) President Lula 2nd term (Lula II), 2007-2010; (c) President Dilma1st term (Dilma I), 2011-2014; (d) President Dilma 2nd term, impeached, and President Term (2015-2018); (e) PresidentBolsonaro, 2019-2022.

Figures 7 and 8 (full data in Tables S1 and S2 in the supplementary material) allows a more detailed description of the temporal evolution (across governmental periods) of the main demographic and epidemiological features of each mosaic. Data differs statistically by region, period, and dimension analyzed (see Table S3).

The Northwest (NWFM), Southwest (SWFM), and North (NOFM) frontier mosaics account for the majority of cases (36.4%, 34.9%, and 27.6%, respectively) from 2007–2023, with their highest concentration between 2007 and 2010. The primary differences between these three mosaics relate to the growth of cases – increasing in the Northwest and declining in Southwest and North – and to race/ethnicity, based on data from 2015–2023: i) NWFM with a much higher and increasing proportion of malaria cases among indigenous population, ii) SWFM also had a significant increase in cases among indigenous population, albeit from a lower baseline than the NWFM, alongside a higher proportion of cases among black individuals (with a decreasing trend), and iii) NOFM had a stable, higher proportion of cases among black individuals. The percentage of the youngest with malaria is proportionally higher at NWFM and SWFM compared to all other mosaics.

In contrast to these three mosaics, *P. falciparum* and mixed malaria infections were proportionally more concentrated in mosaics of very small number of malaria cases: the Northeast (NEFM, especially from 2007–2014) and Southeast (SEFM, 2007–2010). However. in terms of absolute number of cases, NWFM (with increasing numbers) and SWFM stand out. Furthermore, NEFM and SEFM had a lower percentage of autochthonous cases, and NEFM had by far the higher percentage of imported malaria and SEFM the higher percentage of allochthonous malaria. They also showed a higher incidence among men and those aged 15–39, and among white individuals compared to other mosaics. NEFM also had a high percentage among black individuals.

Overall, sex and age showed minor variations across mosaics and periods. From 2015–2023, cases among the indigenous population increased across all combined frontier mosaics, while cases among black individuals decreased.

## DISCUSSION

Mosaics show how the spatiotemporal evolution of mosaics follows the intersection between malaria incidence, distinct occupational profiles related to diverse land use and settlement patterns, forms of human mobility rather than permanent migration, and rural-urban connectivity within a dynamic policy environment. Identifying and understanding local-level dynamics using data on localities show that that malaria is often clustered in specific, localized land-use areas and settlements, and that malaria cases have diminished and become increasingly focal over time.

Regionalization identified areas with distinguishing transmission dynamics. We observe two primary patterns. First, *High-Transmission Frontiers (NWFM, SWFM, NOFM)* are characterized by higher concentration of cases, intense local (*autochthonous*) and *P. vivax* transmission. The NWFM is a critical concern, with an escalating case burden, a disproportionate impact on Indigenous populations, on the youngest, and a rising incidence of *P. falciparum*. While the NOFM and SWFM show an overall decline, the SWFM has experienced a concurrent increase in cases within its Indigenous communities.

Second, in *Low-Transmission Mosaics (NEFM, SEFM*), malaria is shaped by human mobility as shown by higher relative prevalence of allochthonous and imported cases and among men and the most active labor force (15–39). Urban centers act as residential hubs, with individuals—particularly male laborers—likely contracting the disease in remote *garimpos* and indigenous lands and importing it back to urban areas, resulting in positive Net Malaria Mobility (NMM).

Results suggest that a critical epidemiological shift is underway: even as overall cases decline, the *proportion* of imported and allochthonous cases is rising. This is hypothetically linked to changing land use and human mobility; while rural cases have decreased with the stabilization of the agricultural frontier, malaria among indigenous population is increasing and urban centers are becoming more connected to remote transmission hotspots. Spatially, clusters of high malaria density are associated with both positive and negative NMM, exemplified by a 2023 cluster in Roraima that linked high local transmission in the Yanomami Indigenous Territory to high imported malaria in the state capital, Boa Vista.

Malaria risk is highly selective of socioeconomic and occupational profiles. In mosaics with fewer cases (SEFM, NEFM), infections are disproportionately concentrated among men aged 15–39. Conversely, high-transmission mosaics show distinct racial compositions, with higher percentages of Indigenous populations in the NWFM and SWFM. A common trait across all mosaics is that malaria has increased among Indigenous populations.

Crucially, these spatiotemporal dynamics do not occur in a political vacuum. Frontier malaria, as a territorial expression of local processes mediated by policy, was significantly impacted by the flexibilization of environmental laws in Brazil between 2019 and 2022. This policy shift spurred, for example, a surge in illegal mining on indigenous lands, consolidating a significant transmission frontier linking the Yanomami Indigenous Territory to urban localities like Boa Vista—a direct consequence of political decisions shaping the social epidemiology of the disease. It is likely that shifts in frontier malaria mosaics occurred around this period, and especially after 2015 with the stall and slight increase in malaria particularly in *garimpos* and among indigenous populations.

Although the methodological approach is consistent with prior findings on socioenvironmental determinants of malaria, additional studies are needed to explore other forces influencing the modern Amazon frontier. For example, future work should examine the significance of social and mobility networks as a mechanism of malaria diffusion [19, 34], and climate change as a driver of observed malaria trends. Another research area is the impact of disproportionate population distributions in certain mosaics on incidence analysis. For example, even in mosaics like the NFWM with a stable gender balance, temporal changes in the absolute number or demographic profile of the exposed population could account for shifts in incidence.

We discuss evidences on how distinct periods of federal governance may influence policy factors relevant to malaria transmission. While we do not aim to attribute transmission dynamics to specific policy levers at any administrative scale, future research should examine potential synergies and conflicts among policy levers operating across federal, state, and municipal levels. The five mosaics illustrate characteristic patterns associated with malaria transition; nonetheless, each mosaic may comprise multiple subregional or local mosaics, reflecting the influence of municipal- or state-level policy levers on specific ecological determinants that affect malaria etiology, along with environmental and socioeconomic factors.SIVEP-malaria database is a valuable, high-resolution data source for the Amazon, even in remote areas.

Our method achieved nearly 100% spatial identification of cases and localities from 2007–2023 by using auxiliary municipality and land-use data. However, SIVEP has limitations for local-level studies. First, geographic coordinates can be inaccurate or misassigned, especially in isolated mosaics or areas with illegal activities. This is problematic for intra-municipal studies requiring precision, though less so for municipal-level analysis which aggregates localities of residence and likely infection and hidden their spatial inaccuracies or no identification. Second, difficult site access can lead to underreporting, particularly in areas like *garimpos*. While severe cases are reliably reported, this, combined with spatial inaccuracies, may create spatially selective data biases in some mosaics. Third, a key data limitation is that coordinates are point estimates and do not represent the actual area (polygons) of localities. This is acceptable for analyzing connectivity and social networks between communities [19, 34], but not for our approach. We therefore employed Kernel Density Estimation to model the territory of malaria transmission. For research directly linking land use or population density to incidence, using actual areas rather than points would necessitate an additional, more complex spatial identification method. Finally, this research is limited to the socio-environmental drivers of spatiotemporal malaria transmission. Consequently, our data and models do not incorporate factors related to vector ecology or human-vector interactions which may affect the geographical shifts of infected mosquitoes.

In conclusion, the frontier malaria mosaic theory is able to expand the restrictive frontier malaria model centered on agricultural expansion, roads, and migration and describe contemporary frontiers defined by complex occupational profiles, human mobility, and diverse land uses (urban, rural, indigenous, illegal mining) within shifting policy contexts. Most studies using aggregated data obscure critical local dynamics involving land use, mobility, socioeconomic conditions, and parasite circulation—a key limitation as transmission becomes increasingly focal. Malaria burden, combined with poverty and unsustainable land use, drives inequality, disproportionately harming younger and Indigenous populations. Our findings underscore the need for local-level surveillance to reach these groups, reduce vulnerability, and promote health equity.

## METHODS

### Study setting.

The Brazilian Legal Amazon spans 5,015,068 km^2^ (58.9% of Brazil’s territory) [48]. [41]. It includes 722 municipalities across nine states: the entire territories of Amazonas, Pará, Acre, Rondônia, Roraima, Amapá, Tocantins, and Mato Grosso, plus a portion of Maranhão [48].

### Data.

Municipal health systems collect malaria case data for SIVEP-Malaria. Public data, available since 2007, includes parasite types, patient demographics, types of parasites and clinical tests and treatments and type of localities of residence and likely infection (e.g., urban, rural, *garimpo* etc). *Locality* is a point location, such as a neighborhood, village, mining site, among others. SIVEP-malaria registers 3,578,079 malaria cases between 2007 and 2023.

Since many localities in the database lack coordinates, we developed a four-step imputation method:

1. *Data Collection*: Gathering spatial data databases with coordinates or spatial features (points, lines, and polygons) from official sources like the National Address Register (CNEFE-IBGE), Brazilian Indigenous Foundation (FUNAI), waterway maps from the National Water Agency (ANA) and the National Institute of Colonization and Agrarian Reform (INCRA).
2. *Standardization*: Normalizing locality names in the malaria dataset (i.e., lowercase letters, no accents, no prepositions, etc.).
3. *Matching*: Using textual similarity algorithms (RapidFuzz library) [49] to match standardized names to the collected spatial databases.
4. *Integration*: Adding the newly geolocated localities to those already with coordinates in SIVEP.

SIVEP-Malaria classifies localities into 44 types, which we aggregated into four analytical categories: Urban, *Garimpos*, Indigenous, and Rural (including rural settlements from agrarian reform). Following this definition, dataset included 160,179 localities between 2007 and 2023.

### Modelling strategy.

We analyzed the spatiotemporal evolution of frontier malaria in three steps. First, descriptive statistics detail the increased spatial coverage of malaria cases achieved by our geolocation method and present the key variables for analysis. Among these, we created a variable to measure how population mobility between infection and residence localities can foster local transmission. Based on the concept of Malaria Mobility Networks [19], the Net Malaria Mobility (NMM) at locality *i* with land use *L* at time *t* is defined as:

1 $$NMM_{i,t}^{L}=\left(\sum_{j\neq i}^{J}IM_{ji,t}^{L}\right)-AM_{i,t}^{L}$$

$IM_{ji,t}^{L}$ represents *Imported Malaria* (individuals living in a locality *i* in Brazil who had likely infection in a different locality, *j*, in Brazil or abroad) and $AM_{i,t}^{L}$ represents Autochthonous Malaria (individuals infected in the same locality, *i*, of residence). If positive, $NMM_{i,t}^{L}$ suggests that malaria in a locality is mostly driven by the mobility of infected people, and if negative, that malaria is mainly local. Values closer to zero means both the absence of malaria cases in locality *i* or a balance between imported and autochthonous cases.

Second, we identified spatiotemporal clusters of localities with varying concentrations of malaria cases. Annualized indicators enabled cross-period comparison. We then used Kernel Density Estimation (KDE) with a quartic function to create a smoothed, continuous spatial surface. A 100 km bandwidth and 1 km cell size were applied, weighting nearby points more heavily to estimate the probability density^1^. Thirdly, we created a regular hexagonal grid for local estimation of the NMM indicator, with 50km between centroids.

Finally, we propose a regionalization using a clustering technique to delineate homogeneous regions with similar local-level malaria infection characteristics. The SKATER method [49–50] (Spatial ‘K’luster Analysis by Tree Edge Removal) create homogeneous, contiguous regions (or mosaics) that maximize between-cluster variance and minimize within-cluster variance based on a multidimensional vector of *n* variables standardized using Z-scores. Input variables that distinguish each mosaic included the percentage of cases by land use (urban, rural, *garimpo*, indigenous), and the proportion of autochthonous and *P. falciparum* cases. In order to prevent the formation of excessively small regions resulting from the territory’s dispersed spatial pattern - namely, vast areas with small populations - a constraint was established in the algorithm requiring that, at each iteration, regions must comprise at least 10% of the total population of the Legal Amazon.

The SKATER method builds a graph where nodes are hexagonal grid cell centroids and edges connect contiguous cells. It then prunes weaker connections to produce a Minimum Spanning Tree (MST), retaining only the strongest links between similar nodes (Assunção, Lage, & Reis, 2002). Regionalization proceeds by iteratively cutting the edge with the greatest dissimilarity, thereby partitioning space into progressively more homogeneous clusters.

Dissimilarity between any two spatial units (*i* and *k*) is quantified using the Euclidean distance across *m* variables relevant to malaria infection:

2 $$D_{i,k}=\sqrt[{2}]{\sum_{j=1}^{m}{\left(x_{ij}-x_{kj}\right)}^{2}}$$

The MST is built by computing edge dissimilarities and retaining only minimal-cost connections linking all units. Pruning a predefined number of edges—set by the desired number of regions—produces homogeneous clusters (Assunção, Lage, & Reis, 2002). For example, to form two clusters, the algorithm calculates the sum of squared deviations for the variables from the global mean of all units in the tree:

3 $$Total\;sum\;of\;squares\;\left(SST\right)={\sum_{j=1}^{m}\sum_{i=1}^{n}\left(x_{ij}-x_{-j}\right)}^{2}$$

The algorithm iteratively deletes edges from the MST. Each removal partitions the tree into two clusters, evaluated by the within-cluster sum of squared deviations (SSW); summing SSW across clusters gives SSWR, where lower values indicate greater homogeneity. Edge removal cost is SST minus SSWR. The edge with the highest cost is pruned, producing the most homogeneous clusters at that step.

We implemented the SKATER method in GeoDa^®^ using a hexagonal grid of 2,537 cells (50 km between centroids). Variables—location of infection (urban, mining, Indigenous, rural), proportion of *Plasmodium falciparum* cases, and percentage of autochthonous cases—were normalized to Z-scores, with Euclidean distance defining graph edges. A final constraint required each cluster to contain at least 10% of the Legal Amazon’s total population (2022 census), yielding five clusters (Table 2). Although the regionalization explained only 9.1% of total variance—due to high heterogeneity and sparse data in unpopulated areas—the outcome is satisfactory. The model successfully accommodates the region’s challenging population distribution, reflects its complex settlement history, and identifies distinct internal patterns offering valuable avenues for future research.

## Supplementary Material

This is a list of supplementary files associated with this preprint. Click to download.

• SupplementaryInformation.docx

## Figures and Tables

**Table 1:** Improvements in the spatial identification of localities and of malaria cases by categories of localities of residence, 2007–2023

Variables	Types of Localities of Residence					
	Total	Urban	Garimpo	Indigenous	Rural	Not
						identified
Localities registered	162,840	9,223	2,134	6,557	144,190	736
in SIVEP (n, 2007–2023, %)	100.0%	100.0%	100.0%	100.0%	100.0%	0.0%
Localities with coordinates	11,516	1,769	180	2,151	7,416	0
in SIVEP (n, 2007–2023, %)	7.1%	19.2%	8.4%	32.8%	5.1%	0.0%
Improved spatial identification of	148,663	7,410	1,950	4,397	134,906	0
localities (n, 2007–2023, %)	91.3%	80.3%	91.4%	67.1%	93.6%	0.0%
Total localities with spatial	160,179	9,179	2,130	6,548	142,322	0
identification (n, 2007–2023, %)	98.4%	99.5%	99.8%	99.9%	98.7%	0.0%
Malaria cases registered	3,601,061	1,270,672	94,731	538,267	1,675,079	22.312
in SIVEP (n, 2007–2023, %)	100.0%	100.0%	100.0%	100.0%	100.0%	100.0%
Malaria cases with coordinates	2,581,540	1,028,557	65,553	456,075	1,031,355	0
in SIVEP (n, 2007–2023, %)	71.7%	80.9%	69.2%	84.7%	61.6%	0.0%
Improved spatial identification	996,539	241,522	29,164	82,192	643,661	0
of malaria (n whole period, %)	27.7%	19.0%	30.8%	15.3%	38.4%	0.0%
Total cases with spatial	3,578,079	1,270,079	94,717	538,267	1,675,016	0
identification (n, 2007–2023, %)*	99.4%	100.0%	100.0%	100.0%	100.0%	0.0%

Source: Elaborated from SIVEP-Malaria dataset

* There are 670 cases not identified by types of localities of residence (urban, garimpo, rural and indigenous). Adding this number to the 22,312 cases not found in the localities database totals 22,982 cases, which represents 0.6% of the overall amount.

**Table 2:** Frontier malaria regions (clusters)

Regions	likely local of infection				Parasite especies	Autochtonous (%)
	Urban	Garimpo	Indigenous	Rural	falciparum	
Northeastern (NEFM)	7,4%	11,3%	2,5%	25,0%	25,7%	15,5%
Northern (NOFM)	4,8%	17,0%	5,2%	59,8%	13,3%	57,4%
Northwestern (NWFM)	3,8%	1,0%	21,7%	34,5%	9,2%	45,7%
Southeastern (SEFM)	6,0%	8,9%	4,7%	16,2%	9,8%	13,2%
Southwestern (SWFM)	7,1%	4,3%	16,1%	54,8%	11,2%	59,1%

## Footnotes

1. For the determination of the bandwidth, the mean distance criterion was applied. A distance matrix was generated, and it was found that, for the 1,000 nearest neighbors, the mean distance was 105.7 km, and 59.7% of the neighbors were located less than 100 km away. Additionally, visual tests indicated that values close to 100 km produced results that were neither overly smoothed nor overly dispersed throughout the Legal Amazon.
